# In Vitro Anti-Glioblastoma Activity of a Novel Pt(IV)-Ganoderic Acid A Conjugate

**DOI:** 10.3390/ijms27062760

**Published:** 2026-03-18

**Authors:** Ludovica Gaiaschi, Fabrizio De Luca, Stefano Artin Serapian, Emma Lugli, Federica Maraschi, Arianna Bini, Daniele Merli, Maria Grazia Bottone

**Affiliations:** 1 Laboratory of Cell Biology and Neurobiology, Department of Biology and Biotechnology “L. Spallanzani”, University of Pavia, 27100 Pavia, Italy; ludovica.gaiaschi@unipv.it (L.G.); fabrizio.deluca@unipv.it (F.D.L.); emma.lugli01@universitadipavia.it (E.L.); 2 Department of Chemistry, University of Pavia, 27100 Pavia, Italy; stefanoartin.serapian@unipv.it (S.A.S.); federica.maraschi@unipv.it (F.M.); arianna.bini01@universitadipavia.it (A.B.); daniele.merli@unipv.it (D.M.)

**Keywords:** glioblastoma, platinum-based chemotherapy, phyto-derived compound, drug resistance

## Abstract

Glioblastoma is the most aggressive primary malignant tumor of the central nervous system in adults, with a poor prognosis and high resistance to conventional therapies. Platinum drugs like cisplatin are effective but limited by systemic toxicity, poor blood–brain barrier penetration, and resistance. Natural compounds are increasingly studied for their anticancer potential and ability to enhance existing therapies. Based on this rationale, we designed Pt(IV)Ac-GA, a novel platinum(IV) complex obtained by conjugating cisplatin with ganoderic acid A, a triterpenoid from *Ganoderma lucidum* known for anticancer and immunomodulatory effects. The compound was synthesized, structurally characterized, and showed high stability and favorable pharmacokinetics. In vitro, Pt(IV)Ac-GA strongly reduced the viability of U251 and T98G glioblastoma cells while sparing normal astrocytes. It triggered apoptosis, cell cycle arrest, impaired migration, and increased sensitivity to ferroptosis and mitochondrial dysfunction. These results highlight Pt(IV)Ac-GA as a promising candidate to overcome current limitations in glioblastoma treatment.

## 1. Introduction

Glioblastoma (GB) is the most common and aggressive primary malignant tumor of the central nervous system (CNS) in adults. It represents nearly 15% of all primary CNS tumors and accounts for approximately 50% of all malignant primary CNS tumors. Despite multimodal treatment approaches (including maximal surgical resection, radiotherapy, and chemotherapy with the DNA-alkylating agent temozolomide (TMZ)), GB remains invariably fatal, with a median survival of only 12–15 months following diagnosis [1]. The highly infiltrative nature of GB, its profound inter- and intra-tumoral heterogeneity, the presence of glioma stem cells, and the effect of the altered blood–brain barrier (BBB) all contribute to its resistance to conventional therapies. In this context, the search for alternative or adjunct therapeutic agents remains a critical goal [2].

Among the compounds investigated for their potential in GB therapy, platinum-based chemotherapeutics have attracted considerable attention. Cisplatin (cis-diamminedichloridoplatinum(II), CDDP), in particular, exerts potent cytotoxic effects primarily by forming DNA adducts that interfere with replication and transcription, ultimately leading to apoptosis. However, its clinical use in GB has been limited due to systemic toxicity [3,4,5,6] and acquired resistance mechanisms that limit CDDP efficacy [7,8,9].

To overcome these limitations, novel cisplatin derivatives with improved profiles are being developed. These next-generation platinum(II) and platinum(IV) complexes aim to enhance selective tumor uptake, reduce systemic toxicity, and bypass resistance mechanisms, while retaining or augmenting the cytotoxic potency of the parent compound. Octahedral Pt(IV) complexes offer an efficient strategy to combine a traditional Pt(II) moiety (e.g., CDDP) with a second bioactive molecule in the axial position, allowing for synergistic or additive effects and ameliorated pharmacokinetic properties. These prodrugs are selectively reduced in the hypoxic tumor microenvironment, releasing both the cytotoxic Pt(II) species and the co-ligated drug [10,11], representing an effective anticancer therapeutic strategy and, according to recent studies, a potential new approach for cancer screening [12].

In recent years, complementary and integrative medicine approaches have gained increasing attention in oncology [13,14,15,16]. Natural compounds derived from plants and fungi exhibit a broad spectrum of biological activities, including anti-inflammatory, immunomodulatory, and anticancer properties, that may enhance therapeutic efficacy and improve patient quality of life. Mushroom-derived compounds have demonstrated promising anticancer and immunomodulatory effects both in vitro and in vivo [17,18,19]. Species such as *Ganoderma lucidum*, *Hericium erinaceus*, *Lentinula edodes*, *Grifola frondosa*, and *Cordyceps sinensis* contain bioactive molecules (e.g., polysaccharides, triterpenoids, and phenolic compounds) that, according to previous in vitro and in vivo studies, exert antiproliferative effects, modulate oxidative stress, and may synergize with standard chemotherapeutics [20,21,22,23].

Ganoderic acid A (GA), particularly, exhibits broad antitumor activities across various cancers. In glioblastoma cultured cells, GA inhibits proliferation, migration, and invasion by suppressing the PI3K/AKT/mTOR pathway, promoting apoptosis via increased Bax and caspase-3 and decreased Bcl-2 expression [24]. Beyond glioblastoma, GA exhibits neuroprotective and antitumor activities in models of other cancers. In triple-negative breast cancer, GA reduces cancer cell viability in vitro by downregulating the JAK2/STAT3 pathway. Notably, GA derivatives have been shown to modulate MDM2 in zebrafish breast cancer models, with an overall pro-apoptotic effect [25,26]. In lung carcinoma, GA suppresses autophagy, increasing tumor cell sensitivity to cisplatin [27]. Hepatocellular carcinoma GA, in a mouse model, modulates the tumor microenvironment by regulating tumor-associated macrophage polarization through inhibition of CSF1R, enhancing antitumor immunity and suppressing tumor progression [28]. In gallbladder cancer, GA potentiates cisplatin cytotoxicity, reducing stemness markers, suggesting its ability to reduce cancer stem cell properties [29]. In prostate cancer, GA suppresses androgen-independent cancer cell growth via the AKT/GSK-3β/β-catenin pathway, inhibiting tumorigenesis and cell proliferation [30]. Similarly, it resulted in targeting β-catenin signaling and inducing apoptosis in pancreatic cancer cells [31]. As observed in tumor cells, where GA appears to promote cellular stress states that may facilitate the activation of programmed cell death pathways, its anti-inflammatory effects have been consistently validated in healthy zebrafish models, further supporting its dual role in modulating cell fate depending on the physiological context [32].

Building on this rationale, a novel Pt(IV) complex, Pt(IV)Ac-GA, has been developed by covalently linking platinum(IV) diacetate to ganoderic acid A (GA), a triterpenoid isolated from *Ganoderma lucidum* with established anticancer, antioxidant, and anti-inflammatory properties [33,34]. This innovative conjugate holds promise to advance GB therapy by synergistically enhancing cytotoxicity, overcoming cisplatin resistance, improving tumor selectivity, and minimizing systemic toxicity through targeted activation in the hypoxic tumor environment.

## 2. Results

### 2.1. Pt(IV)Ac-GA Synthesis and Characterization

**Figure 1:**
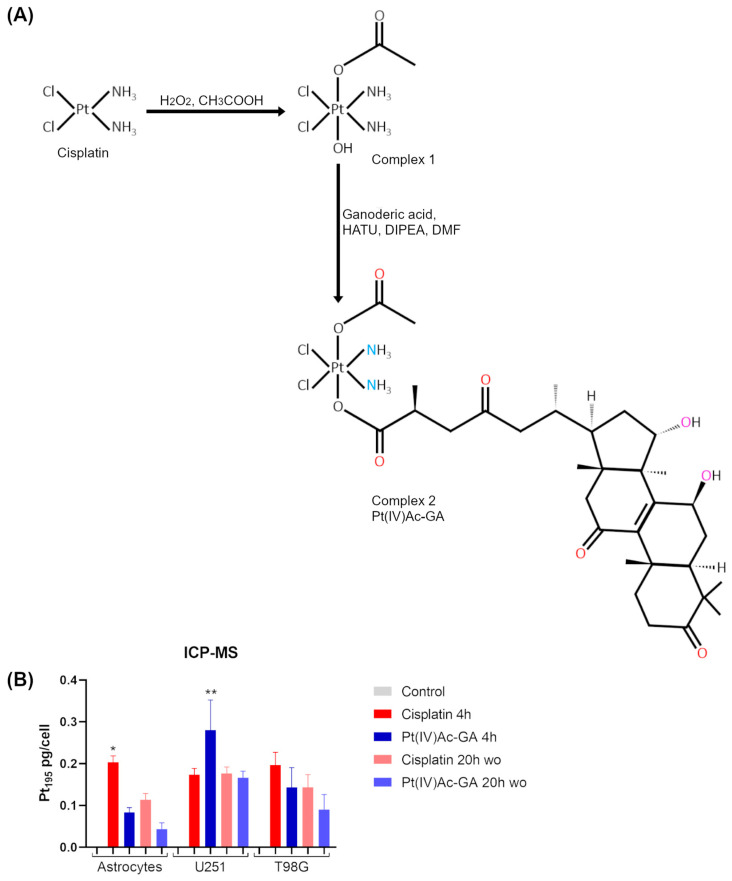
Synthesis and cellular uptake of the Pt(IV)Ac-GA complex. (**A**) Synthesis of the octahedral Pt(IV) complex (OC-6-44)-acetate(β-ganoderate)diamminedichloridoplatinum(IV) [Pt(IV)Ac-GA]. HATU: Hexafluorophosphate Azabenzotriazole Tetramethyl Uronium; DIPEA: N,N-diisopropylethylamine; DMF: dimethylformamide. In the structural formula of Pt(IV)Ac-GA, hydrogen bond acceptors are shown in red, hydrogen bond donors in blue, and atoms that can act as both in purple. (**B**) Platinum195 amount measured in human astrocytes, U251 and T98G, after treatment with 100 μM of cisplatin (CDDP) or Pt(IV)Ac-GA for 4 h and after 20 h of washout (wo) in clean media. Histogram showing the mean Pt195 amount expressed as pg/cell ± SD, significance of differences: * *p* < 0.05, ** *p* < 0.01, where * indicates the difference in each treatment versus the Control condition (n = 3).

The final compound, (OC-6-44)-acetate(β-ganoderate)diamminedichloridoplatinum(IV) [Pt(IV)Ac-GA], was successfully synthesized by covalently linking platinum(IV) monohydroxy monoacetate to ganoderic acid A (GA) (Figure 1A). The product was isolated as a faint yellow solid with a yield of 97%. Characterization by ICP-OES indicated a platinum content of 21.8%, in good agreement with the theoretical value of 22.3% calculated for the molecular formula C_32_H_52_Cl_2_N_2_O_9_Pt. Electrospray ionization mass spectrometry (Supplementary Materials S1) confirmed the expected molecular ion at *m*/*z* 872.2619 [M − H]^−^ (calcd. 872.2625), with a mass error of −1 ppm. The 1H, 13C NMRs are reported in Supplementary Materials S1. Thus, we generated an octahedral Pt(IV) complex combining the cisplatin moiety with a second bioactive molecule in the axial position, namely ganoderic acid A. As shown in Figure 1A, the newly synthesized compound bears ganoderic acid A in one axial position, while an inert acetate group occupies the opposite axial site. The central platinum core and the equatorial ligands remain identical to those of cisplatin. Structural analysis of the molecule indicated the presence of five hydrogen bond acceptors, including two carboxylate oxygen atoms coordinated to Pt(IV) and three carbonyl oxygen atoms. Additionally, two heavy-atom hydrogen bond donors were identified as two ammine (NH_3_) nitrogen atoms. Also, two hydroxyl oxygen atoms, which can serve as both donors (via H) and acceptors (via O lone pairs), were recognized.

The compound’s lipophilicity, a parameter for pharmacokinetics, was evaluated by predicting the *logP* value at 298.15 K [35]. DFT optimization, as already described, at the M11/MWB60/6-311+G(*d*,*p*) level in implicit *n*-octanol and water estimated a −4.91 kJ mol^−1^ difference in Gibbs free energy of solvation in favor of *n*-octanol. Plugged into equation (1) above, this yields a predicted *logP* value of +0.860 (Supplementary Materials S2). Overall, these data confirm the successful synthesis, structural integrity, and favorable lipophilicity profile of Pt(IV)Ac-GA, supporting its suitability for cellular studies. On the basis of these chemical and physicochemical features, the cellular uptake and retention of platinum were next investigated.

To evaluate the cellular uptake and retention of platinum in different cell lines following exposure to 100 μM of cisplatin (CDDP) or the Pt(IV) compound Pt(IV)Ac-GA, ICP-MS analysis was performed in viable (thereby assuming preserved membrane integrity) astrocytes, U251, and T98G cells. In astrocytes, treatment with CDDP for 4 h led to a significant increase in Pt uptake compared to untreated cells (*p* = 0.0217), with a Pt accumulation increase (0.203 ± 0.015 pg/cell versus 0.000 ± 0.000). On the other hand, Pt(IV)Ac-GA did not significantly differ from the Control (0.083 ± 0.012, *p* > 0.9999). Following washout, both compounds showed a decrease in intracellular Pt levels, with CDDP retainment (0.113 ± 0.015) corresponding to a 44% decrease compared to uptake, and Pt(IV)Ac-GA retainment (0.043 ± 0.015) showing a 48% reduction, although these changes were not statistically significant (*p* > 0.9999). In U251, only Pt(IV)Ac-GA 4 h treatment showed a significant increase in Pt accumulation compared to the Control (0.280 ± 0.072 versus 0.000 ± 0.000, *p* = 0.0073). CDDP uptake did not significantly differ from the Control (0.173 ± 0.015, *p* = 0.2611). After washout, Pt levels in the Pt(IV)Ac-GA-treated cells decreased to 0.167 ± 0.015, corresponding to a 40% reduction, while CDDP-treated cells maintained levels (0.177 ± 0.015). Finally, in T98G, no comparisons reached statistical significance. The CDDP 4 h treatment yielded the highest Pt accumulation (0.197 ± 0.031), while Pt(IV)Ac-GA uptake was lower (0.143 ± 0.047), and its retention dropped to 0.090 ± 0.036, indicating a 37% loss after washout. CDDP retention also declined to 0.143 ± 0.031 (a 27% reduction) (Figure 1B). Taken together, these results indicate that Pt(IV)Ac-GA displays a cell-line-dependent uptake and retention profile that differs from cisplatin, suggesting distinct intracellular handling mechanisms, in particular, between healthy and tumoral cells. These differences in platinum accumulation prompted an evaluation of cytotoxic and pro-apoptotic effects.

### 2.2. Cytotoxic and Pro-Apoptotic Effects

**Figure 2:**
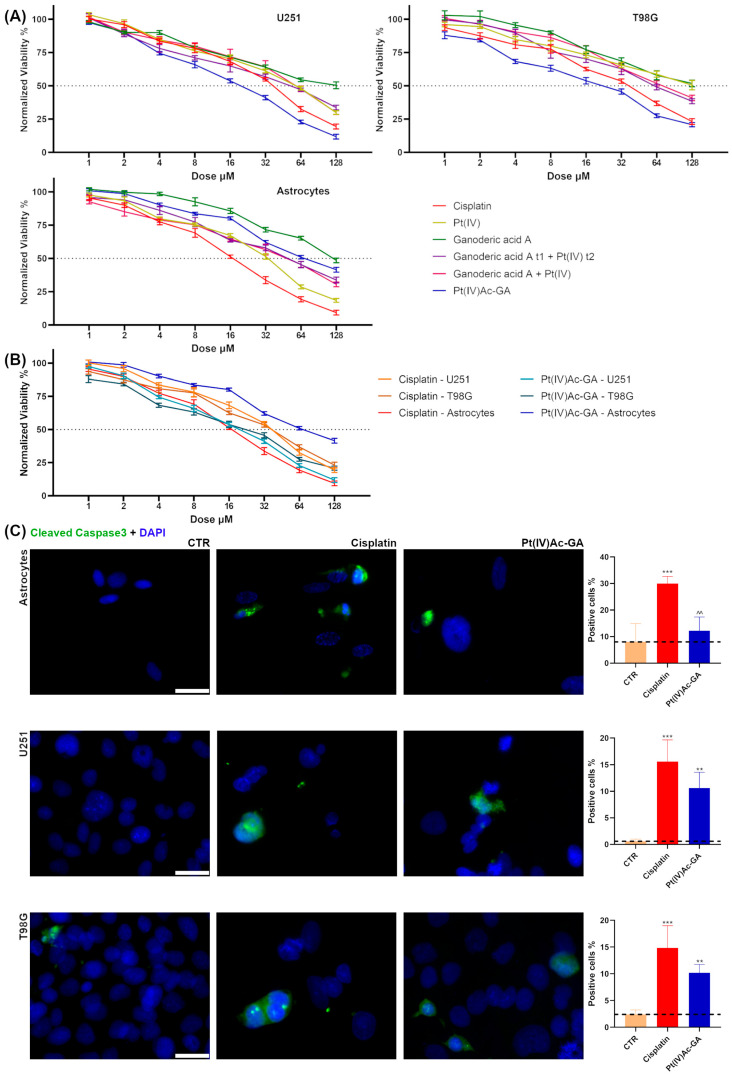
Pt(IV)Ac-GA reduces viability and induces apoptosis in glioblastoma cells. (**A**) Curve representing viability of U251, T98G, and human astrocytes obtained with MTT assay after 48 h acute exposure to increasing concentrations (0–128 μM) of cisplatin, Pt(IV) precursor compound, ganoderic acid A, the combination of ganoderic acid A and the Pt(IV) compound (either simultaneously or with a 6 h GA pre-treatment), and the Pt(IV)Ac-GA conjugate. The cell viability is expressed as a percentage relative to the untreated Control cells (n = 3). (**B**) Curve comparing viability of U251, T98G, and human astrocytes obtained with MTT assay after 48 h acute exposure to increasing concentrations (0–128 μM) of cisplatin and the Pt(IV)Ac-GA conjugate. The cell viability is expressed as a percentage relative to the untreated Control cells (n = 3). (**C**) Immunocytochemical detection of cleaved Caspase 3 (green) in Control cells, and in cells exposed to CDDP (40 μM) or Pt(IV)Ac-GA (20 μM) for 48 h; human astrocytes, U251 and T98G were tested. DNA was counterstained with Hoechst 33258 (blue). Bar 40 μm. Histograms showing the mean percentage of cleaved Caspase 3^+^ immunolabeled cells ± SD, significance of differences: **/^^ *p* < 0.01, *** *p* < 0.001, where * indicates the difference in each treatment versus the Control condition and ^ indicates the difference in Pt(IV)Ac-GA versus cisplatin (n = 4).

The MTT assay performed after 48 h of acute treatment revealed differential cytotoxic effects across glioblastoma cell lines (U251 and T98G) and healthy astrocytes (Figure 2A, Table 1). Cisplatin significantly reduced cell viability in all cell types, with a reduction in viability of 50% in astrocytes already at low doses (16 µM), and with less efficacy in T98G and U251, for which the IC50 on cell viability was reached at 38 µM and 41 µM, respectively. The Pt(IV) precursor exhibited moderate toxicity in U251 (IC50 at 62 µM) and astrocytes (IC50 34 µM), but was poorly effective in T98G cells, reaching the reduction in viable cells of 50% at 128 µM. Ganoderic acid A alone showed negligible cytotoxicity (IC50 at 128 µM in U251; 128 µM in T98G; 128 µM in astrocytes). When administered prior to Pt(IV) (sequential treatment), ganoderic acid A appeared to sensitize both glioma cell lines, with a reduction of 50% viability at 54 µM in U251 and 61 µM in T98G. The simultaneous co-treatment yielded a similar effect, with IC50 obtained at 64 µM in U251 and 73 µM in T98G µM. Notably, the conjugated compound Pt(IV)Ac-GA demonstrated the highest cytotoxicity in both tumor lines, with the IC50 at 20 µM in U251; 25 µM in T98G), while showing reduced toxicity in healthy astrocytes (IC 50 at 66 µM), highlighting its potential selective anticancer activity. Calculation of the selectivity index (SI = IC50 in astrocyte/IC50 in glioblastoma cells) revealed that Pt(IV)Ac-GA displayed the highest tumor selectivity, with SI values of 3.3 in U251 and 2.6 in T98G (versus cisplatin’s SI < 1), indicating preferential cytotoxicity toward glioblastoma cells compared with healthy astrocytes (Figure 2B, Table 1).

Importantly, ganoderic acid A alone was essentially ineffective at the concentrations tested, exhibiting minimal cytotoxic activity across all cell types. Given the low toxicity of ganoderic acid at the concentrations relevant to this study and in this experimental setting, and considering that the literature reports data at much higher doses [24], subsequent experiments therefore focused on the newly generated conjugated compound and its parent molecule.

Immunofluorescence analysis of cleaved caspase-3, a known apoptotic marker (Figure 2C), revealed a significant increase in the percentage of positive cells following cisplatin 40 μM treatment compared to the Control (CTR) in all three cell lines. In astrocytes, the percentage of cleaved caspase-3 positive cells increased from 8.02 ± 6.97% (CTR) to 29.99 ± 2.66% with cisplatin (*p* = 0.0006), corresponding to a 274% increase. Pt(IV)Ac-GA 20 μM treatment induced a non-significant increase to 12.20 ± 5.18% (*p* = 0.5217). In U251 cells, cisplatin caused a strong rise from 0.59 ± 0.40% to 15.59 ± 4.06% (*p* = 0.0001; +2559%), while Pt(IV)Ac-GA significantly increased cleaved caspase-3 positivity to 10.63 ± 2.96% (*p* = 0.0022; +1714%). Similarly, in T98G cells, cisplatin raised the percentage from 2.38 ± 0.85% to 14.83 ± 4.16% (*p* = 0.0002; +523%), and Pt(IV)Ac-GA induced a significant increase to 10.18 ± 1.58% (*p* = 0.0057; +327%). A statistically significant difference between cisplatin and Pt(IV)Ac-GA was observed only in astrocytes (*p* = 0.0025), while in U251 and T98G cells, the differences between treatments were not significant. Overall, these findings demonstrate that Pt(IV)Ac-GA combines enhanced antitumor efficacy with reduced toxicity toward healthy cells, indicating improved therapeutic selectivity. To determine whether reduced viability was associated with impaired proliferation and cell-cycle progression, proliferation markers and DNA content were analyzed.

### 2.3. Inhibition of Proliferation and Cell-Cycle Alterations

**Figure 3:**
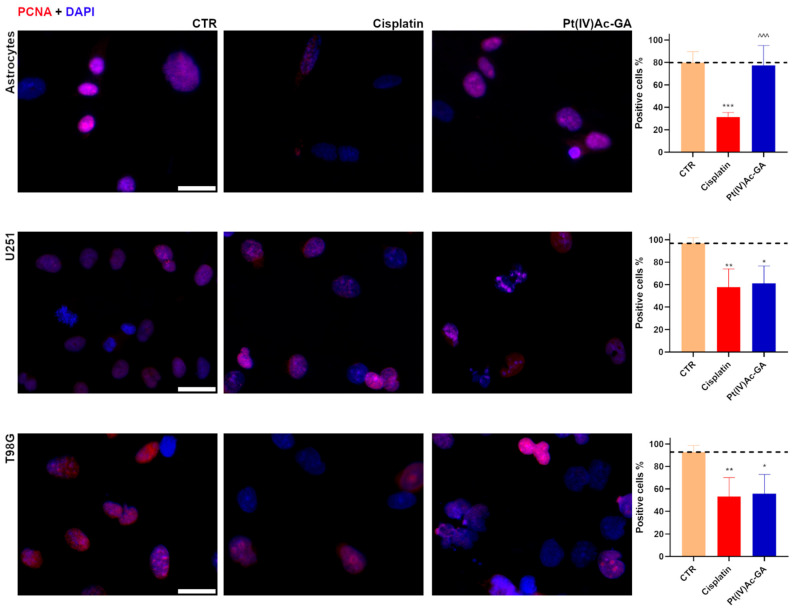
Pt(IV)Ac-GA suppresses cell proliferation marker PCNA in glioblastoma cells. Immunocytochemical detection of PCNA (red) in Control cells, and in cells exposed to CDDP (40 μM) or Pt(IV)Ac-GA (20 μM) for 48 h; human astrocytes, U251 and T98G were tested. DNA was counterstained with Hoechst 33258 (blue). Bar 40 μm. Histograms showing the mean percentage of PCNA^+^ immunolabeled cells ± SD, significance of differences: * *p* < 0.05, ** *p* < 0.01, ***/^^^ *p* < 0.001, where * indicates the difference in each treatment versus the Control condition and ^ indicates the difference in Pt(IV)Ac-GA versus cisplatin (n = 4).

Quantification of PCNA-positive cells (Figure 3) revealed a significant reduction in proliferative activity following cisplatin treatment in all three cell lines. In astrocytes, the percentage of PCNA-positive cells decreased from 79.85 ± 9.67% in the Control to 31.25 ± 4.17% after cisplatin 40 μM treatment (*p* = 0.0007), corresponding to a 61% reduction. In contrast, treatment with Pt(IV)Ac-GA 20 μM did not significantly alter PCNA levels (77.48 ± 17.57%, *p* = 0.9569), and a significant difference between cisplatin and Pt(IV)Ac-GA was observed (*p* = 0.001). In U251 cells, PCNA positivity dropped from 96.86 ± 4.92% (CTR) to 57.82 ± 16.28% with cisplatin (*p* = 0.0063; −40%), and to 61.19 ± 15.50% with Pt(IV)Ac-GA (*p* = 0.0106; −37%). No significant difference was detected between the two treatments (*p* = 0.9319). Similarly, in T98G cells, cisplatin significantly reduced the percentage of PCNA-positive cells from 92.77 ± 5.88% to 53.21 ± 16.80% (*p* = 0.0086; −43%), and Pt(IV)Ac-GA caused a comparable reduction to 55.86 ± 16.95% (*p* = 0.0127; −40%), again with no significant difference between the treatments (*p* = 0.9626).

**Figure 4:**
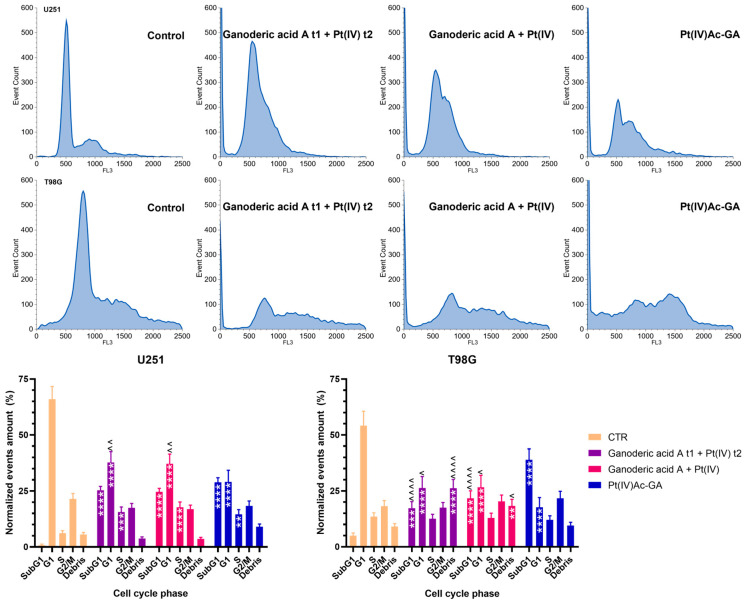
Pt(IV)Ac-GA induces glioblastoma cell death and cell-cycle perturbation. Cytofluorimetric histograms of U251 and T98G DNA content after PI staining in control cells, and in cells exposed to Pt(IV)Ac-GA or the combination of ganoderic acid A and the Pt(IV) compound (either simultaneously or with a 6 h GA pre-treatment) (20 μM) for 48 h; U251 and T98G were tested. The histograms below show the percentage of events in each phase of the cell cycle ± SD in the two lines involved, significance of differences: ^ *p* < 0.05, **/^^ *p* < 0.01, *** *p* < 0.001, ****/^^^^ *p* < 0.0001, where * indicates the difference in each treatment versus the control condition and ^ indicates the difference in Pt(IV)Ac-GA versus the combination of ganoderic acid A and the Pt(IV) compound (n = 3).

Flow cytometry analysis of cell-cycle distribution after 48 h of treatment at 20 μM for each compound (Figure 4) showed pronounced cell-cycle alterations and clear apoptotic signatures in both U251 and T98G glioblastoma cell lines. In U251 cells, all treatment conditions, sequential (Ganoderic acid A t1 + Pt(IV) t2), simultaneous (Ganoderic acid A + Pt(IV)), and conjugated (Pt(IV)Ac-GA), led to a significant accumulation in the subG1 phase, with values rising from 0.77 ± 0.47% in the Control to 25.36 ± 1.73%, 24.61 ± 1.58%, and 28.89 ± 2.06%, respectively. The marked enrichment of the subG1 population following Ganoderic acid A and Pt(IV) or Pt(IV)Ac-GA treatment reflects extensive DNA fragmentation, consistent with severe genomic damage and irreversible commitment to apoptotic cell death. These treatments also induced a pronounced decrease in the G1 phase population (from 66.01 ± 5.62% in the Control to ~37% with the first two treatments and 29.09 ± 5.08% with the conjugate), while increasing the S phase fraction, suggesting cell cycle arrest or progression disturbance. The G2/M population remained relatively stable. Notably, Pt(IV)Ac-GA treatment led to the highest number of high-DNA content events or possible debris (9.02 ± 1.24%).

In T98G cells, the increase in subG1 was even more striking, particularly following Pt(IV)Ac-GA treatment (38.98 ± 4.82% vs. 5.02 ± 1.18% in the Control), indicating robust induction of apoptosis. Sequential and simultaneous treatments also elevated subG1 to 17.31 ± 2.94% and 21.69 ± 3.37%, respectively. These changes were accompanied by a marked reduction in G1 phase cells (from 54.20 ± 6.41% in the Control to ~26–27% in treated samples) and only minor fluctuations in S and G2/M phases. Interestingly, the sequential combination resulted in a substantial increase in the high-DNA/debris region (26.27 ± 3.87%), potentially reflecting increased cell disintegration or polyploidy.

These results indicate that Pt(IV)Ac-GA effectively suppresses proliferation and induces cell-cycle perturbations preferentially in tumor cells. Overall, the prominent accumulation of cells in the subG1 fraction following Pt(IV)Ac-GA treatment provides strong evidence of DNA damage-driven apoptotic cell death, rather than a reversible cytostatic effect. Given the evidence of apoptotic cell death, the subcellular distribution of PARP1, which reflects differential involvement in nuclear DNA damage response or mitochondrial stress signaling, was next examined to gain mechanistic insight.

**Figure 5:**
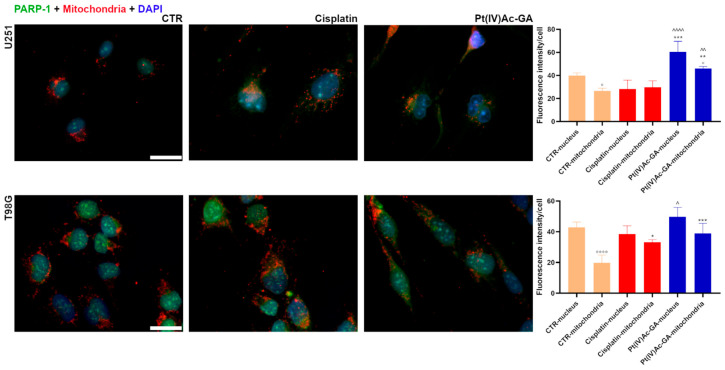
Pt(IV)Ac-GA alters PARP1 subcellular localization. Immunocytochemical detection of PARP1 (green) and mitochondria (red) in control cells, and in cells exposed to CDDP (40 μM) or Pt(IV)Ac-GA (20 μM) for 48 h; U251 and T98G were tested. DNA was counterstained with Hoechst 33258 (blue). Bar 40 μm. Histograms showing the mean fluorescence intensity per cell ± SD, significance of differences: */° *p* < 0.05, **/^^ *p* < 0.01, *** *p* < 0.001, ^^^^/°°°° *p* < 0.0001, where * indicates the difference in each treatment versus the control condition, ^ indicates the difference in Pt(IV)Ac-GA versus cisplatin, and ° indicates the difference in nucleus versus cytoplasm (n = 4).

Analysis of PARP1 fluorescence intensity in nuclear and mitochondrial compartments (Figure 5) revealed distinct subcellular redistribution patterns in U251 and T98G glioblastoma cells in response to treatment. In U251 cells, nuclear PARP1 signal decreased from 39.91 ± 2.36 (CTR) to 28.16 ± 7.86 following cisplatin treatment (*p* = 0.0780; −29%), while it markedly increased to 60.52 ± 9.09 with Pt(IV)Ac-GA (*p* = 0.0008 vs. CTR; +52%). Mitochondrial PARP1 signal remained similar between control cells (IF 26.63 ± 2.42) and cisplatin-treated cells (IF 29.79 ± 5.59), while an increase to 45.98 ± 1.82 has been measured in Pt(IV)Ac-GA samples (*p* = 0.0015 vs. CTR; +73%).

In T98G cells, nuclear PARP1 levels remained statistically unchanged following cisplatin treatment (IF 42.94 ± 3.38 in CTR vs. 38.52 ± 5.39; *p* = 0.8021) and Pt(IV)Ac-GA treatment (IF 49.74 ± 6.08; *p* = 0.4133). In mitochondria, both treatments significantly elevated PARP1 intensity compared to the Control (IF 19.76 ± 5.13): the signal rose to 33.18 ± 1.71 with cisplatin (*p* = 0.0134; +68%) and to 39.03 ± 6.44 with Pt(IV)Ac-GA (*p* = 0.0004; +98%). This redistribution supports the involvement of PARP1-associated apoptotic pathways in the response to Pt(IV)Ac-GA. Because mitochondrial function is closely linked to redox balance, markers of oxidative and inflammatory stress were subsequently assessed.

### 2.4. Disruption of Redox Homeostasis

**Figure 6:**
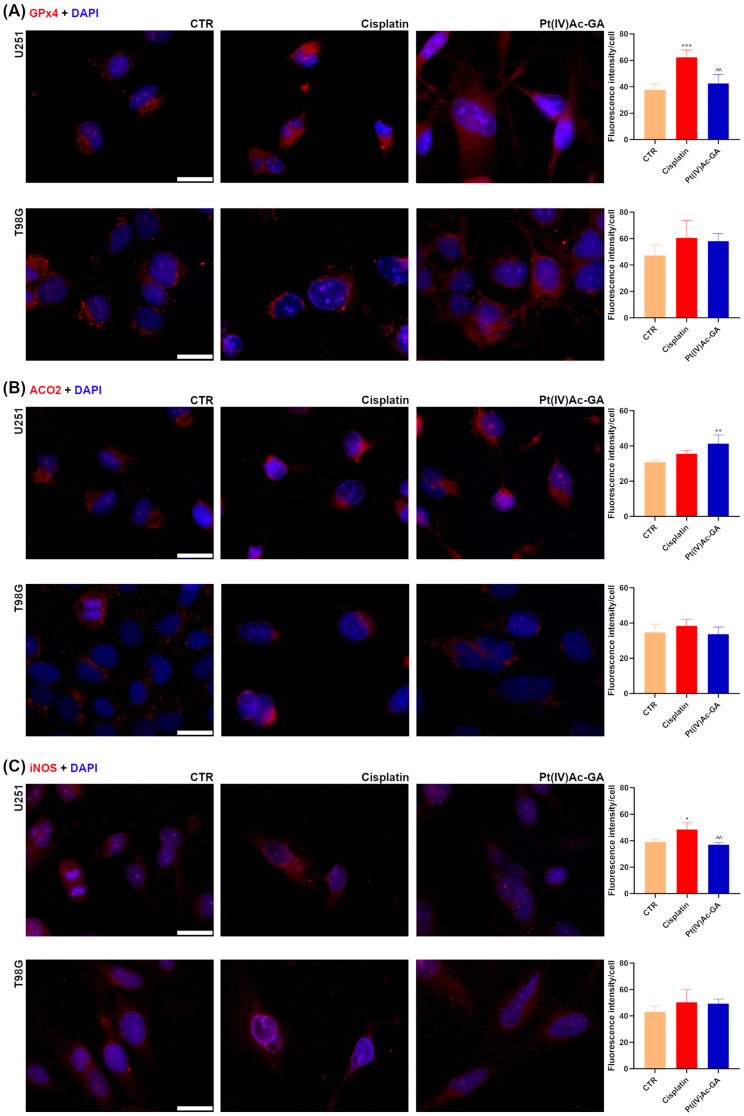
Pt(IV)Ac-GA modulates redox and inflammatory markers. Immunocytochemical detection of GPx4, ACO2 or iNOS (red) in control cells, and in cells exposed to CDDP (40 μM) or Pt(IV)Ac-GA (20 μM) for 48 h; U251 and T98G were tested. DNA was counterstained with Hoechst 33258 (blue). Bar 40 μm. Histograms showing the mean fluorescence intensity per cell ± SD, significance of differences: * *p* < 0.05, **/^^ *p* < 0.01, *** *p* < 0.001, where * indicates the difference in each treatment versus the control condition, and ^ indicates the difference in Pt(IV)Ac-GA versus cisplatin (n = 4).

Insight into the modulation induced by the new compound was obtained through the analysis of molecular markers relevant to redox homeostasis (Figure 6). In U251 cells, GPX4 levels, defense from lipid peroxidation through the reduction in phospholipid hydroperoxides, markedly increased following cisplatin treatment, from 37.71 ± 4.51 in the Controls to 62.36 ± 5.51 (*p* = 0.0004; +65%). Treatment with Pt(IV)Ac-GA caused only a slight, non-significant increase (42.63 ± 6.78; *p* = 0.4698 vs. CTR), and values remained significantly lower than those induced by cisplatin (*p* = 0.0021). In T98G cells, a similar trend was observed: GPX4 intensity increased with cisplatin (47.17 ± 8.24 in CTR vs. 60.55 ± 13.34; *p* = 0.2039), while Pt(IV)Ac-GA induced a milder, non-significant elevation (58.10 ± 5.70; *p* = 0.3729 vs. CTR). Interestingly, a diffuse GPX4 staining pattern, rather than the punctate signal, emerged in the samples exposed to Pt(IV)Ac-GA, suggesting widespread lipid peroxidation.

In U251 cells, only the new compound treatment significantly increased the levels of ACO2, a mitochondrial enzyme of the tricarboxylic acid cycle whose activity is highly sensitive to oxidative stress, making it a functional indicator of mitochondrial redox status, compared to the Control (CTR: 30.38 ± 1.17; cisplatin: 35.57 ± 1.85, *p* = 0.1364; Pt(IV)Ac-GA: 41.33 ± 4.96, *p* = 0.0027). On the contrary, in T98G cells, no significant changes in ACO2 levels were registered after the treatments (CTR: 34.70 ± 4.38; cisplatin: 38.44 ± 3.69, *p* = 0.4259; Pt(IV)Ac-GA: 33.75 ± 4.02, *p* = 0.9404).

In U251 cells, cisplatin treatment led to a significant increase in iNOS levels, responsible for the production nitric oxide, involved in oxidative and nitrosative stress responses, compared to Control (CTR: 39.20 ± 2.24; cisplatin: 48.63 ± 5.01, *p* = 0.0121, +24%), while Pt(IV)Ac-GA exposure left baseline levels of iNOS (37.01 ± 1.72, *p* = 0.6989), result statistically significant compared cisplatin-induced modification (*p* = 0.0058). Once again, in T98G cells, iNOS intensity increased non-significantly after both cisplatin (50.46 ± 9.45; *p* = 0.2762) and Pt(IV)Ac-GA (49.35 ± 3.47; *p* = 0.3810) compared to the Control (43.12 ± 4.21).

These data suggest that Pt(IV)Ac-GA perturbs redox homeostasis through mechanisms distinct from cisplatin, potentially contributing to its antitumor activity.

Finally, the impact of Pt(IV)Ac-GA on glioblastoma cell motility was evaluated to assess effects on aggressive cellular behavior.

### 2.5. Suppression of Cell Migration

**Figure 7:**
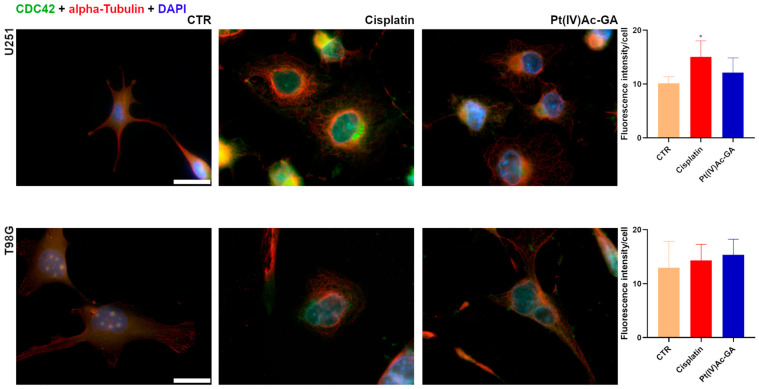
Pt(IV)Ac-GA disrupts cytoskeletal and CDC42 signaling. Immunocytochemical detection of CDC42 (green) and α-Tubulin (red) in control cells, and in cells exposed to CDDP (40 μM) or Pt(IV)Ac-GA (20 μM) for 48 h; U251 and T98G were tested. DNA was counterstained with Hoechst 33258 (blue). Bar 40 μm. Histograms showing the mean fluorescence intensity per cell ± SD, significance of differences: * *p* < 0.05, where * indicates the difference in each treatment versus the control condition (n = 4).

Quantification of CDC42 fluorescence intensity revealed distinct responses to treatment in U251 and T98G glioblastoma cell lines (Figure 7). In U251 cells, CDC42, a small Rho GTPase that regulates cytoskeletal dynamics and promotes cell migration, significantly increased from 10.14 ± 1.23 in the Control to 15.04 ± 3.00 after cisplatin exposure (*p* = 0.0465; +48%), while Pt(IV)Ac-GA treatment led to a moderate and not statistically significant elevation (12.16 ± 2.72; *p* = 0.4978 vs. CTR), suggesting a more limited activation of this small GTPase.

In contrast, T98G cells exhibited no statistically significant changes in CDC42 intensity following either treatment. Fluorescence values increased from 12.97 ± 4.89 in control cells to 14.31 ± 2.98 with cisplatin and 15.37 ± 2.86 with Pt(IV)Ac-GA, but the differences did not reach statistical significance (*p* = 0.8676 and *p* = 0.6435 vs. CTR, respectively).

**Figure 8:**
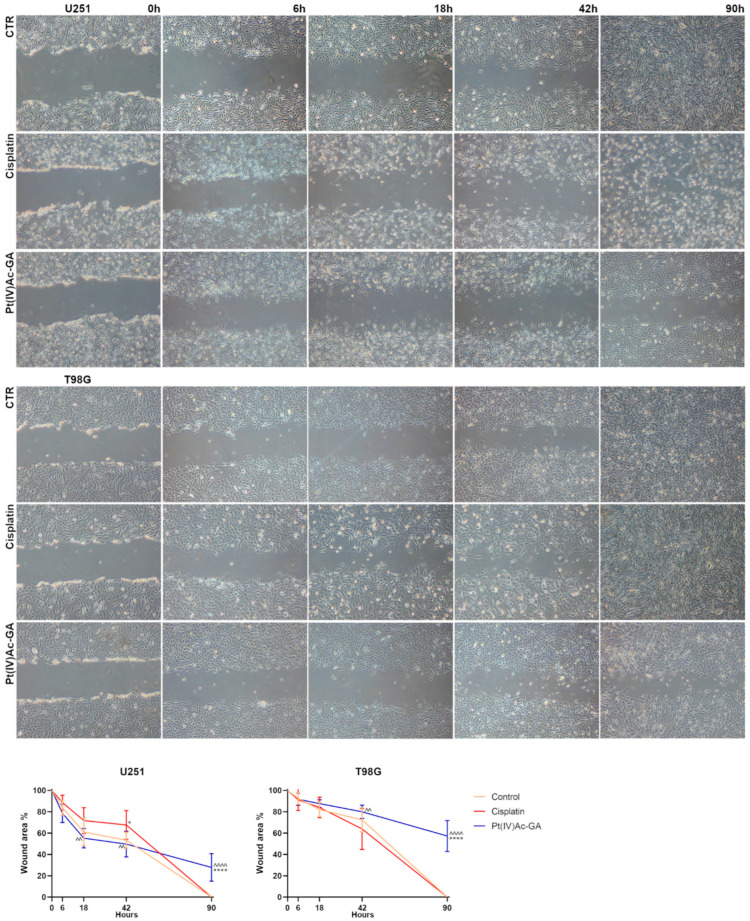
Pt(IV)Ac-GA inhibits glioblastoma cells’ migration. Wound healing scratch assay (4× magnification) on control cells, and in cells exposed to CDDP (8 μM) or Pt(IV)Ac-GA (4 μM) for 48 h; U251 and T98G were tested. The graphs below show the extent of scratch closure expressed as the percentage ± SD, with respect to the initial scratch area (t0), of open scratch area at a distance of 6, 18, 42 and 90 h in the two lines involved, significance of differences, * *p* < 0.05, ^^ *p* < 0.01, ****/^^^^ *p* < 0.0001, where * indicates the difference in each treatment versus the control condition, and ^ indicates the difference in Pt(IV)Ac-GA versus cisplatin (n = 5).

Wound healing was assessed using a scratch assay (Figure 8). In U251 cells, all groups showed a progressive reduction in wound area over time. At the different time points (t1-t2), CDDP and Pt(IV)Ac-GA treatment did not show significant variation from the control condition; at t3, Pt(IV)Ac-GA reduced wound closure (49.68 ± 9.13%) compared to CDDP (67.61 ± 13.51%), with the difference being statistically significant (*p* = 0.0041). At t4, both the Control and CDDP-treated cells reached complete wound closure, whereas Pt(IV)Ac-GA-treated cells maintained 27.79 ± 12.87% of the wound area unclosed. This difference was significant when comparing Pt(IV)Ac-GA to both the Control and CDDP (*p* < 0.0001 for both comparisons). In T98G cells, the wound area remained larger in Pt(IV)Ac-GA-treated cells compared to the other groups at all the timepoints. At t3, Pt(IV)Ac-GA-treated cells showed a significantly higher wound area than those treated with CDDP (79.98 ± 6.29% vs. 63.91 ± 19.16%; *p* = 0.0041), although no significant differences were observed between the Control and either treatment. At t4, both the Control and CDDP groups reached complete wound closure, while Pt(IV)Ac-GA-treated cells retained 57.24 ± 14.51% of the wound area unclosed. This difference was statistically significant when compared to both the Control and CDDP groups (*p* < 0.0001). In this light, Pt(IV)Ac-GA not only reduces glioblastoma cell viability and proliferation but also limits migratory potential, reinforcing its profile as a multifunctional anticancer candidate.

## 3. Discussion

The recent literature has highlighted how natural product (NP)-conjugated platinum complexes can achieve synergistic antitumor activity through multi-target mechanisms, reduced systemic toxicity, and an improved ability to overcome platinum resistance. These features directly address several well-recognized limitations of current glioblastoma (GB) therapies, including poor tumor selectivity, adaptive resistance, and limited engagement of non-canonical cell death pathways. In this framework, NP–Pt(IV) conjugates have emerged as particularly attractive next-generation platinum prodrugs, capable of integrating rational metal-based design with the pleiotropic biological activity of natural compounds [36].

In this context, the newly synthesized compound, (OC-6-44)-acetate(β-ganoderate)diamminedichloridoplatinum(IV) [Pt(IV)Ac-GA], represents a promising advancement in platinum-based therapeutics for glioblastoma. This Pt(IV) prodrug was rationally designed by conjugating ganoderic acid A (GA), a triterpenoid from *Ganoderma lucidum*, to a platinum(IV) scaffold while preserving a cisplatin-like equatorial coordination sphere. Recently, there has been interest in ganoderic acid A conjugates, which have demonstrated remarkable selectivity for tumor cells and exhibited strong inhibitory effects also in vivo [26]. Thus, the present work supports GA as a biologically active sensitizing moiety rather than a simple carrier.

Here, the successful synthesis and structural characterization confirmed the high purity and stability of Pt(IV)Ac-GA. Its predicted lipophilicity suggests enhanced membrane permeability, consistent with the observed increased platinum uptake and retention in GB cells. This aspect is particularly relevant in the context of GB therapy, where insufficient intracellular drug accumulation represents a major obstacle to treatment efficacy. For Pt(IV) prodrugs, increased uptake is especially advantageous, as intracellular reduction to active Pt(II) species is a prerequisite for DNA targeting and cytotoxic activity. Notably, Pt(IV)Ac-GA displayed markedly lower uptake in astrocytes compared to glioblastoma cells. This differential accumulation likely reflects reduced metabolic activity and transporter or endocytic engagement in non-malignant cells, resulting in diminished intracellular platinum levels. Such tumor-selective accumulation is a highly desirable property in GB pharmacology, where the therapeutic window is often limited by damage to surrounding healthy brain tissue.

Pharmacokinetically, the new compound meets Lipinski and Veber rules for oral bioavailability, balancing hydrogen bond donors and acceptors. Although oral delivery was not addressed experimentally, these properties further support the rational design of the conjugate as a drug-like molecule rather than a simple carrier system. Future studies investigating the mechanisms of membrane crossing, as well as in vivo biodistribution analyses, would provide important insights into the selective uptake of the compound.

Cell viability assays revealed potent cytotoxic effects of Pt(IV)Ac-GA on U251 and T98G GB cells, while sparing healthy astrocytes, a key improvement over cisplatin, which harms healthy cells. Ganoderic acid A alone showed negligible cytotoxicity but sensitized glioma cells to platinum in sequential and simultaneous treatments, suggesting that its contribution is not limited to altering the chemical or pharmacokinetic properties of the Pt(IV) prodrug. Instead, GA may interfere with intracellular programs such as redox-responsive signaling pathways or the regulation of transporters. This synergizes with prior findings on mushroom compounds enhancing platinum chemotherapy [20,21,37].

In this study, GA alone showed no detectable cytotoxicity at molar concentrations relevant to Pt(IV)Ac-GA treatment, so standalone GA analyses were not pursued. However, independent studies demonstrate that purified GA or Ganoderma-based nutraceuticals can modulate glioblastoma cell survival, redox balance, and chemotherapy responsiveness, reinforcing its function as a biological sensitizer rather than a direct cytotoxin. Ultimately, the biological activity of Pt(IV)Ac-GA transcends a simple additive effect of its components. The covalent incorporation of the GA moiety into the Pt(IV) scaffold generates emergent properties unattainable by the individual agents alone.

Pt(IV)Ac-GA induced apoptosis, elevating cleaved caspase-3 levels in GB cells but not astrocytes, and reduced PCNA proliferation marker in tumoral cells only. This selective activation of apoptosis is consistent with previous studies showing that micotherapy combined with platinum-based chemotherapy markedly upregulates programmed cell death in GB cells and suppresses proliferation markers in GB models and various other tumoral systems [38,39,40]. Cell cycle analysis provided further insights. Pt(IV)Ac-GA induced a pronounced subG1 accumulation in both glioblastoma cell lines, reflecting advanced apoptotic DNA fragmentation. The pronounced apoptotic response is consistent with increased DNA damage, which is expected upon intracellular reduction in the Pt(IV) prodrug and subsequent release of a cisplatin-like Pt(II) species capable of forming DNA adducts. A parallel increase in the G0/G1 fraction was also detected, a pattern fully compatible with the cytostatic activity expected from an alkylating Pt(IV) scaffold. Nevertheless, analyzing earlier time points would be useful to establish whether Pt(IV)Ac-GA directly imposes a cytostatic block before apoptosis is initiated. The subcellular redistribution of PARP1 further supports the pro-apoptotic action of Pt(IV)Ac-GA. Treatment resulted in a significant increase in mitochondrial PARP1 intensity in both U251 and T98G cells. Beyond its canonical role in nuclear DNA repair, PARP1 has emerged as a critical regulator of mitochondrial integrity and cell death decisions in glioblastoma. Mitochondrial PARP1 accumulation has been associated with NAD^+^ depletion, mitochondrial dysfunction, and amplification of apoptotic signaling cascades, particularly under conditions of extensive DNA damage. Thus, the observed redistribution of PARP1 likely reflects a convergence of nuclear DNA damage and mitochondrial stress, reinforcing apoptotic commitment [41].

Redox homeostasis was differentially altered by the two treatments. Cisplatin markedly increased GPX4 expression in both GB cell lines, consistent with its well-established activation of adaptive antioxidant programs that counteract lipid peroxidation and reduce susceptibility to ferroptosis [42]. Notably, cisplatin also favored iNOS accumulation, suggesting elevated NO/RNS signaling. Given that nitric oxide can quench lipid radicals and thereby suppress ferroptotic initiation [43], this increase may contribute mechanistically to the reduced ferroptotic vulnerability observed under cisplatin treatment.

In contrast, Pt(IV)Ac-GA did not trigger GPX4 upregulation; instead, its profile pointed toward a pro-ferroptotic shift. The significant accumulation of ACO2 is particularly relevant: aconitase is a Fe–S enzyme highly sensitive to redox imbalance, and its increase in a context of unchanged GPX4 may reflect mitochondrial stress and impaired antioxidant buffering, conditions that have been associated with heightened ferroptotic sensitivity. This interpretation aligns with recent work showing that platinum-based compounds in combination with natural therapies can promote ferroptotic cell death in cancer models [21].

Overall, the opposite trends in GPX4, ACO2, and iNOS between the two treatments support the hypothesis that Pt(IV)Ac-GA enhances ferroptosis susceptibility, whereas cisplatin activates compensatory antioxidant and nitrosative pathways that blunt ferroptotic execution. Nevertheless, direct measurements of ROS and RNS levels would be required to confirm the proposed redox-driven mechanisms underlying these differential responses.

Notably, Pt(IV)Ac-GA also impaired cell migration, as shown by CDC42 expression reduction in GB cells in comparison to cisplatin-treated cells and wound healing assays, which showed a wound closure markedly delayed, indicating inhibition of motility and migration [20].

Although natural-compound-based therapies have faced skepticism due to suboptimal drug-like properties, limited intestinal absorption associated with high molecular weight or hydrophilicity, and non-specific assay interference [44,45], these considerations, while important to acknowledge, should not impede progress in the field. The present in vitro findings demonstrate that rational chemical integration of a natural bioactive moiety into a metal-based prodrug can overcome many of these limitations, yielding a compound with synergistic antitumor activity. Taken together, these results provide a strong mechanistic and conceptual foundation for advancing Pt(IV)Ac-GA toward in vivo evaluation and for its development in the context of novel biomimetic drug-delivery systems for cisplatin derivatives [46,47].

Rather than serving as a direct comparator to existing GB chemotherapeutics in this exploratory study, Pt(IV)Ac-GA emerges as a strategically designed candidate that addresses known shortcomings of current treatments and warrants further investigation in more complex preclinical models, including dedicated studies on blood–brain barrier permeability and in vivo validation of its antitumor efficacy, toxicity, and pharmacokinetics, in comparison with clinically relevant standards such as temozolomide, to more fully define its therapeutic potential.

## 4. Materials and Methods

### 4.1. Compound Synthesis

Synthesis of complex (OC-6-44)-acetatodiamminedichloridohydroxidoplatinum(IV). For the synthesis of the complex, a known procedure was followed [48,49]. Briefly, cisplatin (100 mg, 0.33 mmol, Sigma-Aldrich Merck Life Sciences, Milan, Italy) was suspended in glacial acetic acid (40 mL), and hydrogen peroxide 50% *w*/*w* (1 mL, 35 mmol) was added dropwise under stirring at room temperature. The reaction was maintained for 3 h until a clear solution was obtained. The mixture was filtered, and the solvent was removed under reduced pressure. The resulting residue was triturated with diethyl ether (10 mL), then treated with 250 µL of water and 4 mL of 99% ethanol. After the addition of 10 mL diethyl ether, the suspension was filtered and washed with diethyl ether (3 × 10 mL). The solid was dried under vacuum at 25 °C for 24 h to yield a pale-yellow solid of (OC-6-44)-acetatodiamminedichloridohydroxidoplatinum(IV) (105 mg, 75% yield). MW: 376.1. ICP-OES: Pt found 51.2%, calc. 51.9% for C_2_H_10_Cl_2_N_2_O_3_Pt. ^1^H NMR (300 MHz, d_6_-DMSO) in line with data from the literature δ: 1.91 (s, 3H, CH_3_), 5.93 (t, J_H–N = 53.0 Hz, J_H–Pt = 53.0 Hz, 6H, NH_3_) ppm. ESI-MS (positive ion mode): *m*/*z* 377.3 [M + H]^+^ (47%), 359.0 [M − OH]^+^ (100%).

Synthesis of Pt(IV)Ac-GA complex. For the synthesis of Pt(IV)Ac-GA, an established procedure was followed [49]. (OC-6-44)-acetatodiamminedichloridohydroxidoplatinum(IV) (150 mg, 0.400 mmol), ganoderic acid A (C_30_H_44_O_7_, (7|A,15|A,25r)-7,15-dihydroxy-3,11,23-trioxolanost-8-en-26-oic acid, 208 mg, 0.402 mmol, Aktin Chemicals, Inc., Chengdu, China), and HATU (Hexafluorophosphate Azabenzotriazole Tetramethyl Uronium, 220 mg, 0.501 mmol) were suspended in anhydrous DMF (dimethylformamide, 4 mL) under dark conditions. After complete dissolution, N,N-diisopropylethylamine (DIPEA, 150 µL, 0.86 mmol) was added, and the reaction mixture was stirred at room temperature for 24 h. The resulting clear solution was concentrated using a rotary evaporator at 55–60 °C, and the remaining DMF was removed under high vacuum at 25 °C for 12 h, affording an orange viscous mass. Purification was achieved by triturating the crude with a dichloromethane–ether mixture, followed by sequential washing with diethyl ether (4 times), 1% aqueous formic acid (3 times), and cold water (2 times). The final product was dried under vacuum overnight at 25 °C to yield a faint yellow solid (340 mg, 97% yield). ICP-OES: Pt found 21.8%, calc. 22.3% for C_32_H_52_Cl_2_N_2_O_9_Pt. ESI-MS (negative mode): *m*/*z* 872.2619 [M − H]^−^ (calc. 872.2625, −1 ppm mass error), S1. 13C, 1H NMR were in line with the expected. The final molecular weight for C_32_H_52_Cl_2_N_2_O_9_Pt is 873.27 g/mol.

### 4.2. Lipophilicity and Solubility Assessment

To computationally predict key physicochemical properties for the new compound, pharmacokinetic, lipophilicity, which influences membrane permeability, and solubility, critical for bioavailability, were assessed. All quantum chemical calculations were performed using the Gaussian 16 software package [50]. The assessment began with a systematic preliminary exploration of conformers and spin states of Pt(IV)Ac-GA and subportions thereof. The procedure to derive *logP* follows recommendations by Nedyalkova et al. [35]: a low-energy conformer of Pt(IV)Ac-GA, constructed following our preliminary investigation, was separately optimized at the M11 [51]/MWB60 [52]/6-311+G(*d*,*p*) level of Density Functional Theory, first in implicit water and then in implicit *n*-octanol, both modeled according to the SMD implicit solvation model [53] as implemented in Gaussian 16. More specifically, in both optimizations, the MWB60 effective core potential and basis set [52] were used to model electrons on the Pt(IV) center (60 core + 14 valence electrons), whereas the 6-311+G(*d*,*p*) basis set was used to treat electrons on all remaining atoms.

The prediction for *logP* is derived using the following formula [35]:

(1) $$logP=-\frac{{\Delta G}_{o/w}}{2.303RT}$$

where *R* is the ideal gas constant in J mol^−1^ K^−1^; *T* is the standard temperature of 298.15 K; and the difference in Gibbs free energy of solvation in *n*-octanol and water Δ*G*_o/w_ is derived via the following subtraction:

(2) $${\Delta G}_{o/w}=G_{octanol}-G_{water}$$

Estimated standard Gibbs free energies of solvation of our complex in *n*-octanol and water at *T* = 298.15 K—respectively, *G_octanol_* and *G_water_*—are directly derived by performing frequency (Hessian) calculations on the optimized structures as per the standard Gaussian 16 implementation. The same frequency calculations also confirm that the two conformers are energetic minima.

All Gaussian 16 calculations, including the preliminary conformational and spin state exploration, are provided electronically in a dedicated repository [https://doi.org/10.19061/iochem-bd-6-557] on the ioChem-BD platform [54].

### 4.3. Cell Culture

Human glioblastoma cell lines, i.e., U251 MG (RRID: CVCL_0021) and T98G (RRID: CVCL_0556), were cultured in 75 cm^2^ culture flasks using Eagle’s Minimal Essential Medium (EMEM) supplemented with 10% fetal bovine serum (FBS), 2% or 1% L-glutamine for U251 or T98G, respectively, 1% sodium pyruvate, 1% non-essential amino acids, and 100 U/mL penicillin–streptomycin. Cells were maintained at 37 °C in a humidified atmosphere containing 5% CO_2_.

Primary cultures of normal human astrocytes (ScienCell Research Laboratories (Carlsbad, CA, USA), Catalog No. #1800), kindly provided by Prof Paolillo Mayra (Department of Drug Sciences, University of Pavia), served as healthy Controls. No Research Resource Identifier (RRID) is currently available for this cell product; these cells are human astrocytes, isolated from the human cerebral cortex and cryopreserved at passage one. Here, normal human astrocytes were used as the non-pathological reference in comparison to the disease-model condition. The absence of an RRID does not compromise the validity of our findings.

Astrocytes were maintained in DMEM-F12 (Dulbecco’s Modified Eagle Medium and Ham’s F-12 Nutrient Mixture) supplemented with 10% FBS, 1% L-glutamine, and 1% penicillin–streptomycin, under identical incubation conditions (37 °C, 5% CO_2_). All reagents were sourced from Celbio S.p.A. and Euroclone S.p.A. (Pero, Milan, Italy).

Cells were plated at a density of 4 × 10^6^ cells in T75 flasks, or at a density of 6 × 10^5^ cells on covers, in 6-well multiwell plates. Once the cells reached an 80% confluence, they were treated at continuous exposure for a duration of 48 h with the compounds under investigation. The concentrations of molecules used were chosen on the basis of the in vitro MTT viability assay as follows.

### 4.4. Platinum Quantification by ICP-MS

Platinum quantification was performed using a single quadrupole inductively coupled plasma–mass spectrometer (SQ-ICP-MS, iCAP RQ Thermo Fisher Scientific, Monza, Italy), equipped with a quartz cyclonic chamber cooled at 3 °C, a MicroMist nebulizer 179 (400 μL/min, Thermo Fisher Scientific, Monza, Italy), a quartz torch, a Ni sampler, skimmer cones, and a QCell pressurized with helium (3 V, KED mode), operated with the ICP-MS Qtegra software 2.8. Standard curves for platinum quantification were prepared with dilutions of a 1000 mg/L standard solution of Pt (Merck, Milan, Italy) at 0.1, 1, 10, 50, 100, and 1000 μg/L with 0.5% *w*/*w* acidified MilliQ water. The isotope selected for platinum detection was Pt_195_. For in vitro analysis, 3 × 10^6^ cells per sample (including U251 and T98G glioblastoma cell lines, as well as primary human astrocytes) were analyzed after treatment with 100 μM cisplatin or 100 μM Pt(IV)Ac-GA conjugate for 4 h in FBS-depleted media to assess platinum uptake. To evaluate platinum retention, a parallel set of samples underwent the same 4 h treatment, followed by a 20 h washout period in drug-free medium. Between the treatment and the drug-free washout period, the flasks were rinsed three times with 1× PBS. After treatment, flasks were rinsed three times with 1× PBS to remove debris or extracellular and loosely bound platinum species, then cells were detached with Trypsin-EDTA 1× (Euroclone S.p.A., Pero, Italy), counted with the Trypan Blue method, pelleted, washed with 1× PBS four times and digested with 0.2 mL of 70% *w*/*w* HNO_3_ (Suprapur, Merck) + 0.2 mL 35% *w*/*w* hydrogen peroxide (Suprapur, Merck, Milan, Italy) for 1 h at 65 °C. Samples were brought to a final volume of 3 mL with MilliQ water and analyzed by ICP-MS.

### 4.5. MTT Assay

Cell viability was evaluated using the MTT assay (3-(4,5-dimethylthiazol-2-yl)-2,5-diphenyltetrazolium bromide; Sigma-Aldrich, Milan, Italy). Cells were seeded at a density of 5000 cells per well in 100 μL of complete medium in 96-well plates and allowed to adhere for 24 h at 37 °C in 5% CO_2_. Following this, the culture medium was replaced with medium containing increasing concentrations (0–128 μM) of the tested compounds. Cells were then incubated for 48 h under standard conditions. At the end of treatment, 10 μL of MTT solution (5 mg/mL in PBS) was added to each well, and plates were further incubated for 3 h at 37 °C to allow viable cells to reduce MTT into insoluble formazan crystals. Subsequently, the medium was carefully removed, and 100 μL of dimethyl sulfoxide (DMSO) was added to each well to solubilize the formazan. Plates were gently shaken for 10 min at room temperature. Absorbance was recorded at 490 nm using an ELx808™ microplate reader (Bio-Tek Instruments, Winooski, VT, USA), with background correction. Experiments were conducted in triplicate, and cell viability was calculated relative to untreated Controls. Cisplatin, Pt(IV) precursor compound, ganoderic acid A, the combination of ganoderic acid A and the Pt(IV) compound (either simultaneously or with a 6 h GA pre-treatment) and the Pt(IV)Ac-GA conjugate were tested across the same concentration range, ensuring comparable molar amounts of the GA and Pt(IV) moieties between the combination treatments and the conjugate.

Compounds were dissolved directly into the culture medium immediately prior to treatment to preserve stability.

### 4.6. Flow Cytometry

To analyze cell-cycle distribution, U251 or T98G cells were seeded in 75 cm^2^ flasks at a density of 4 × 10^6^ cells per flask and subjected to treatment with the combination of ganoderic acid A and the Pt(IV) compound (either simultaneously or with a 6 h GA pre-treatment) or the Pt(IV)Ac-GA conjugate at 20 μM for 48 h. Post-treatment, cells were harvested by trypsinization and washed twice with sterile PBS. Cells were fixed by adding cold 70% ethanol dropwise while gently mixing and incubated for 10 min at room temperature. Fixed cells were washed again in PBS, treated with RNase A (100 U/mL) to remove RNA, and stained with propidium iodide (PI, 50 μg/mL; Sigma-Aldrich Merck Life Sciences, Milan, Italy) for 10 min at room temperature in the dark. Samples were immediately analyzed on a Partec PAS III flow cytometer (Münster, Germany). PI fluorescence was detected using a 610 nm long-pass filter. Data analysis was performed using the free online software Floreada (https://floreada.io/).

### 4.7. Immunofluorescence Analysis

For immunofluorescence staining, U251 cells were seeded on glass coverslips at 6 × 10^5^ cells per coverslip in 6-well plates and cultured until approximately 80% confluence. Cells were then treated for 48 h with cisplatin 40 μM or Pt(IV)Ac-GA 20 μM. Cells were fixed with 4% paraformaldehyde for 20 min at room temperature, followed by post-fixation in 70% ethanol at −20 °C for 24 h. After rehydration in PBS, nonspecific binding sites were blocked by incubating cells in PBS containing 0.2% Tween-20 and 4% bovine serum albumin (BSA). Primary antibodies (listed in Table 2) were applied for 1 h at room temperature. Following washes, cells were incubated with fluorescent secondary antibodies (Alexa 594 or 488 conjugated anti-mouse or anti-rabbit antibody, Alexa Fluor, GeneTex, Irvine, CA, USA, 1:200 dilution) for 45 min in the dark. Nuclear counterstaining was performed with Hoechst 33,258 (0.1 μg/mL) for 5 min. Coverslips were mounted using Mowiol mounting medium and stored protected from light until imaging.

Fluorescent images were acquired using an Olympus BX51 microscope equipped with a MagnaFire digital camera (Olympus, Shinjuku-ku, Tokyo, Japan). Exposure times were optimized and held constant for each marker based on control samples to avoid bias. Quantitative fluorescence intensity per cell measurements were performed using ImageJ software 1.51 (NIH, Bethesda, MD, USA).

### 4.8. Scratch Wound Healing Migration Assay

Cells were seeded at 6 × 10^5^ cells per well in six-well plates to form a confluent monolayer over 12 h, and then treated with each compound at a subtoxic concentration (Cisplatin 8 μM or Pt(IV)Ac-GA 4 μM). A linear scratch was created using a sterile 10 μL pipette tip across the monolayer. Detached cells and debris were removed by washing with PBS. Images of the wound area were acquired immediately after scratching (time 0, t0) and at distances of 6, 18, 42 and 90 h (t1, t2, t3, t4) using the same microscope setup. Wound closure was quantified by measuring the remaining wound area at each time point relative to the initial wound area (t0) using ImageJ software. Data are reported as the percentage of wound area over time.

### 4.9. Statistical Analyses

Data are presented as means ± SD (Standard Deviation), and at least three independent replicates were performed for each experiment. Significance was determined via one-way ANOVA and Tukey’s Multiple Comparison test or Kruskal–Wallis statistic test, as appropriate. Normality of the data was assessed using the Anderson–Darling, D’Agostino and Pearson, Shapiro–Wilk, and Kolmogorov–Smirnov tests. Significance is indicated as follows: (* *p* < 0.05, ** *p* < 0.01, *** *p* < 0.001, **** *p* < 0.0001). Statistical analysis was performed using GraphPad Prism v8.4.0.

## 5. Conclusions

The Pt(IV)Ac-GA conjugate was rationally designed as a combinatorial prodrug, integrating the cytotoxic Pt(IV) scaffold with the bioactive ganoderic acid A moiety within a single molecular entity. The results from toxicity assays and flow-cytometry analyses strongly support a cooperative, rather than merely additive, interaction between the two components. Pt(IV)Ac–GA exhibits enhanced tumor selectivity and antitumor efficacy in glioblastoma models, promoting apoptosis and ferroptosis, disrupting redox homeostasis, and significantly impairing cell migration, while sparing healthy astrocytes. These findings indicate that ganoderic acid A functions not only as a biocompatible axial ligand but also retains biologically relevant activity that amplifies the therapeutic impact of the platinum core. Collectively, this study provides a proof of concept for natural product–platinum prodrugs, demonstrating that rational conjugation of a natural product to a Pt(IV) scaffold can overcome key translational limitations of conventional platinum-based therapies. By simultaneously addressing cytotoxicity, selectivity, redox vulnerability, and migratory potential of glioblastoma cells, Pt(IV)Ac–GA emerges as a promising multi-functional therapeutic candidate, warranting further preclinical investigation.

## Figures and Tables

**Table 1:** Summary of IC50 values for cell viability and corresponding selectivity indices of the analyzed compounds on glioblastoma cell lines (U251, T98G) and healthy astrocytes.

Compound/Treatment	IC50 U251 (µM)	IC50 T98G (µM)	IC50 Astrocytes (µM)	Selectivity Index (U251)	Selectivity Index (T98G)
Cisplatin	41	38	16	0.39	0.42
Pt(IV) precursor	62	128	34	0.56	0.27
Ganoderic Acid A	128	128	128	1.0	1.0
Ganoderic Acid A + Pt(IV) (sequential)	54	61	51	0.94	0.83
Ganoderic Acid A + Pt(IV) (co-treatment)	64	73	49	0.77	0.67
Pt(IV)Ac-GA (conjugate)	20	25	66	3.30	2.64

**Table 2:** Primary antibodies used for immunofluorescence reactions.

Antigen	Antibody Details	Dilution
PCNA	Calbiochem (Merck, Milan, Italy), mouse monoclonal (PC10)	1:150
Cleaved Caspase 3	GeneTex (Prodotti Gianni, Milan, Italy), rabbit polyclonal (cleaved Asp175)	1:100
PARP 1	Cell Signaling Technology (Danvers, MA, USA), rabbit polyclonal	1:100
GPx 4	GeneTex, rabbit polyclonal	1:100
ACO 2	Abcam (Prodotti Gianni, Milan, Italy), mouse monoclonal	1:100
iNOS	GeneTex, rabbit polyclonal	1:100
CDC 42	GeneTex, rabbit polyclonal	1:100
Human autoimmune serum for mitochondria detection [55]	Kindly provided by IRCCS Policlinico San Matteo, Pavia (Italy)	1:200
α-Tubulin	Invitrogen (Thermo Fisher Scientific, Monza, Italy), mouse monoclonal	1:100

## Data Availability

The data that support the findings of this study are openly available in the ioChem-BD platform at https://doi.org/10.19061/iochem-bd-6-557 or in the Supplementary Materials.

## Supplementary Materials

The following supporting information can be downloaded at: https://doi.org/10.19061/iochem-bd-6-557 (ioChem-BD platform) or https://www.mdpi.com/article/10.3390/ijms27062760/s1.
