# Supplementary material for: In Vitro Anti-Glioblastoma Activity of a Novel Pt(IV)-Ganoderic Acid A Conjugate
Source: Int J Mol Sci. 2026 Mar 18;27(6):2760. doi: 10.3390/ijms27062760 (PMC13026249; doi:10.3390/ijms27062760)
Supplement: Supplementary file 1 [file ijms-27-02760-s001.zip › Supplementary material S1.pdf]

Complex Pt - Ganoderic acid A

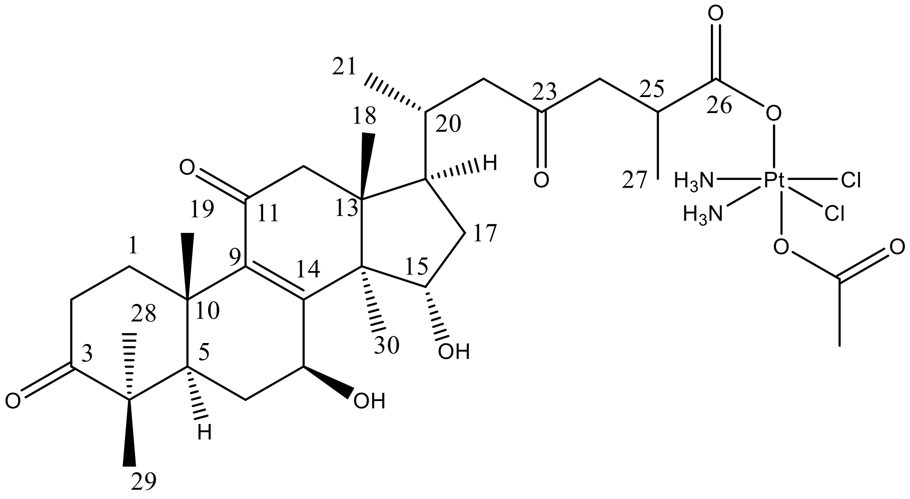

Data are in accordance with literature [Jiang, C., Ji, J., Li, P., Liu, W., Yu, H., Yang, X., … Fan, Y. (2021). New lanostane-type triterpenoids with proangiogenic activity from the fruiting body of Ganoderma applanatum. Natural Product Research, 36(6), 1529–1535. https://doi.org/10.1080/14786419.2021.1898388].

^{1}H:

DMSO-d6, (700MHz) 6.47 (m, 6H, NH3) 5.35 (s, 1H, OH), 4.73-4.66 (m, 1H, H7), 4.62 (s, 1H, OH), 4.53-4.48 (m, 1H, H15), 2.96-2.62 (m, 5H, H24+25+22), 2.48-2.31 (m, 4H, H2) 2.30-2.14 (ABX, 2H, H12), 1.95-1.80 (m, 4H, CH3Pt + H16), 1.78-1.60 (m, 4H, H1+H6), 1.59-1.52 (m, 1H, H20), 1.45-1.40 (qui, 1H, J = 9Hz, H5), 1.26-1.24 (m, 1H, H17), 1.21-1.20 (d, 3H, J = 7Hz, H27), 1.16 (s, 3H H30), 1.14 (s, 3H, H19), 1.03 (s, 3H, H28), 0.98 (s, 3H, H29), 0.79-0.76 (m, 3H, H21);

^{13}C:

DMSO-d6, (175MHz) 216.6 (C3), 209.3 (C23), 190.7 (C11), 178.6 (PtOCO) 177.2 (C26), 161.6 (C8), 139.5 (C9), 71.2 (CH, C15), 67.7 (CH, C7), 54.1 (C14), 52.1 (CH2, C12), 49.5 (CH2, C22), 48.2 (CH, C5), 46.7 (CH, C17), 46.5 (C13), 46.4 (CH2, C24), 46.3 (C4), 37.6 (C10), 36.7 (CH2, C6), 35.6 (CH2, C1), 34.6 (CH, C25), 34.2 (CH2, C2), 32.6 (CH, C20), 29.2 (CH2, C6), 27.2 (CH3, C28), 27.0 (CH3, Pt), 20.9 (CH3, C29), 20.4 (CH3, C21), 19.6 (CH3, C19), 19.2 (CH3, C30), 17.6 (CH3, C18), 17.4 (CH3, C27)

© Gaiaschi et al.

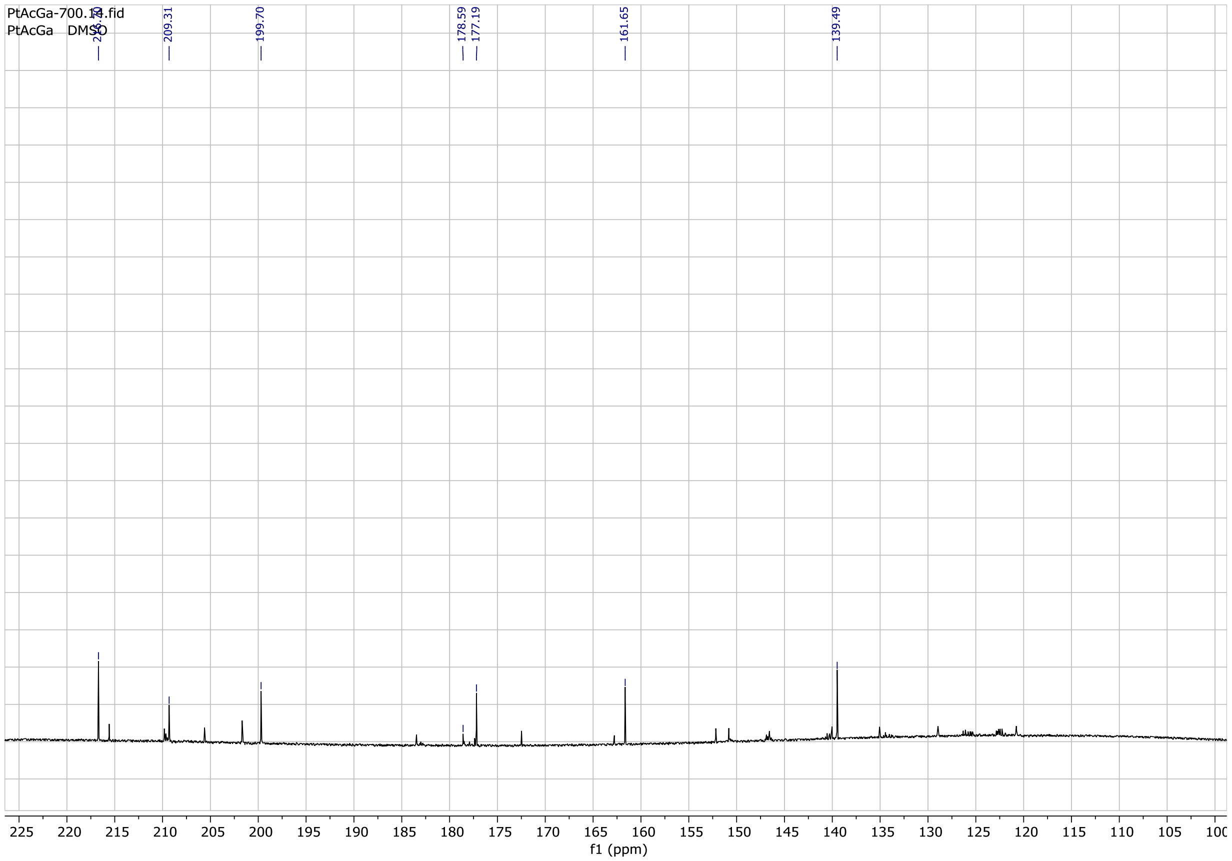

Figure S1-A. ^{13}C NMR spectra, enlargement of the 225-100 MHz range

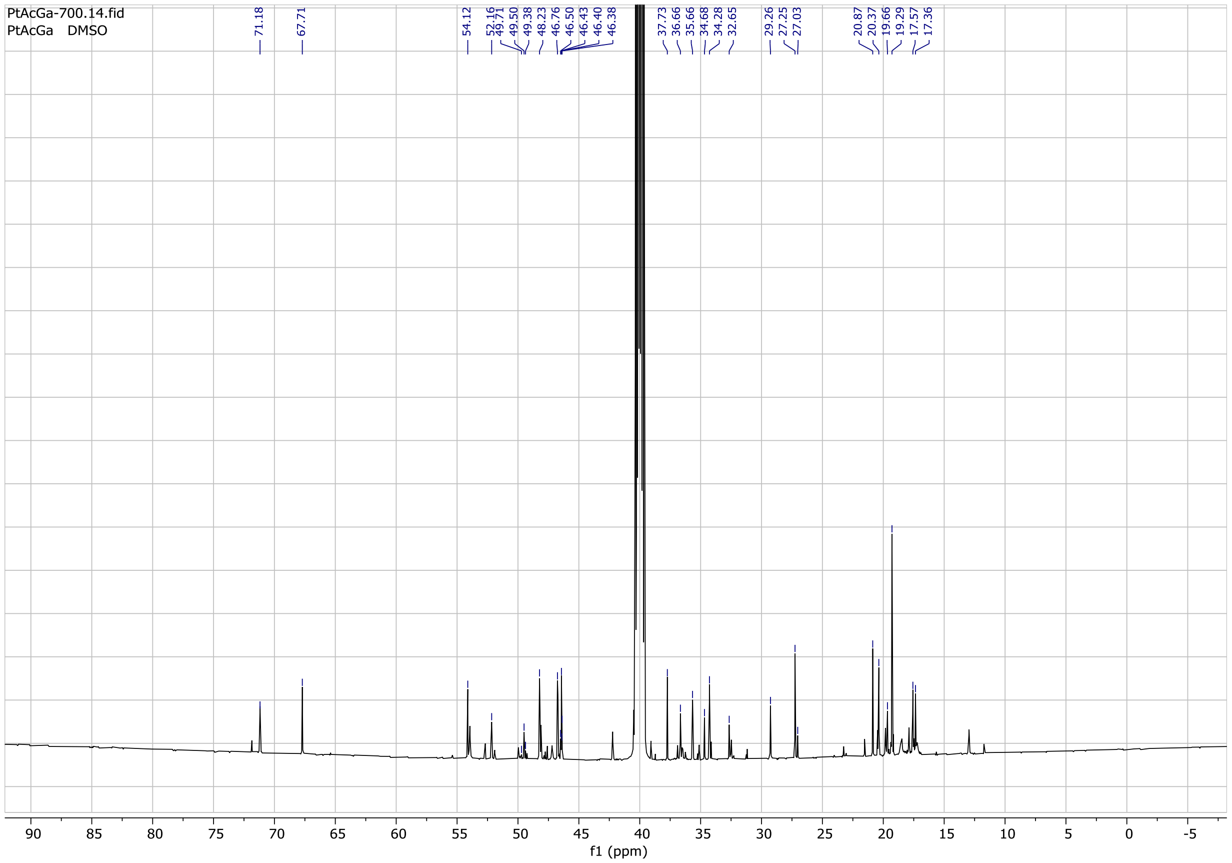

Figure S1-B. ^{13}C NMR spectra, enlargement of the 90-0 MHz range

© Gaiaschi et al.

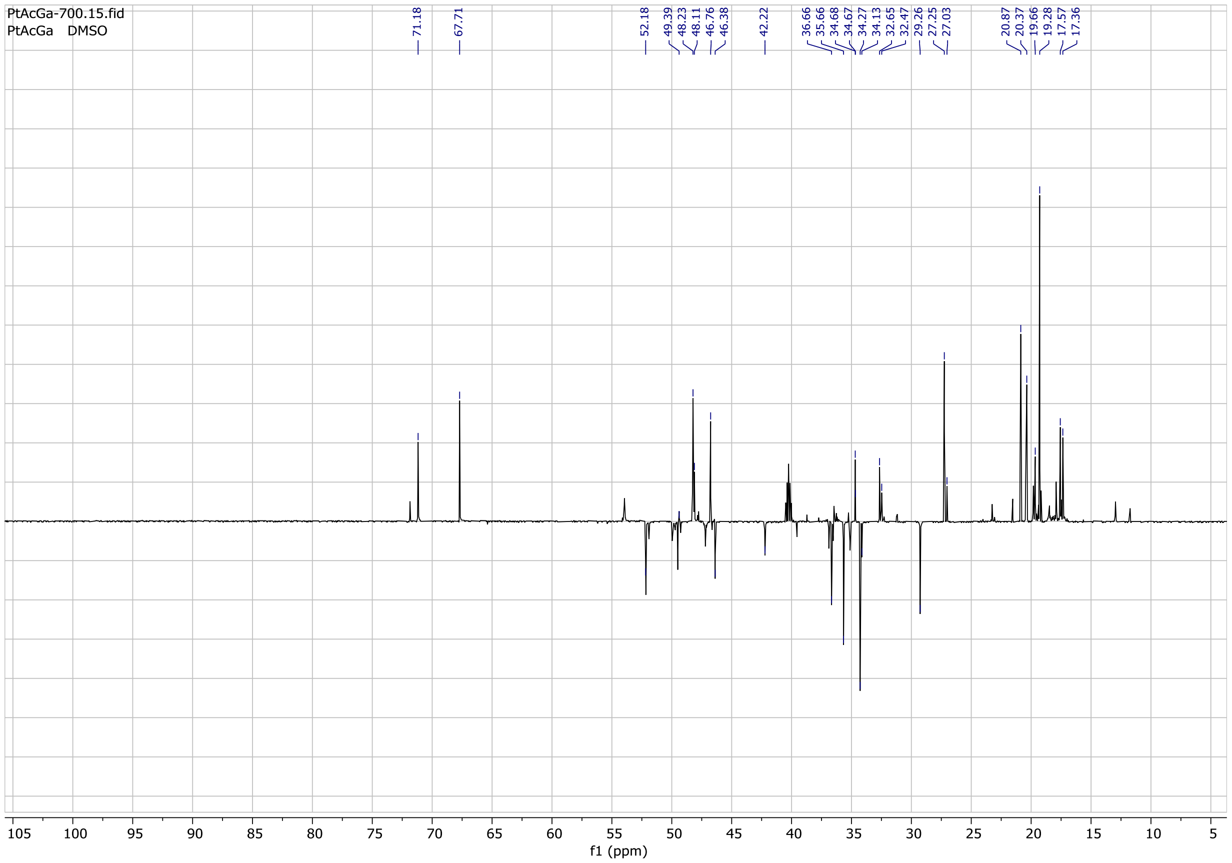

Figure S1-C. ^{13}C NMR DEPT spectra

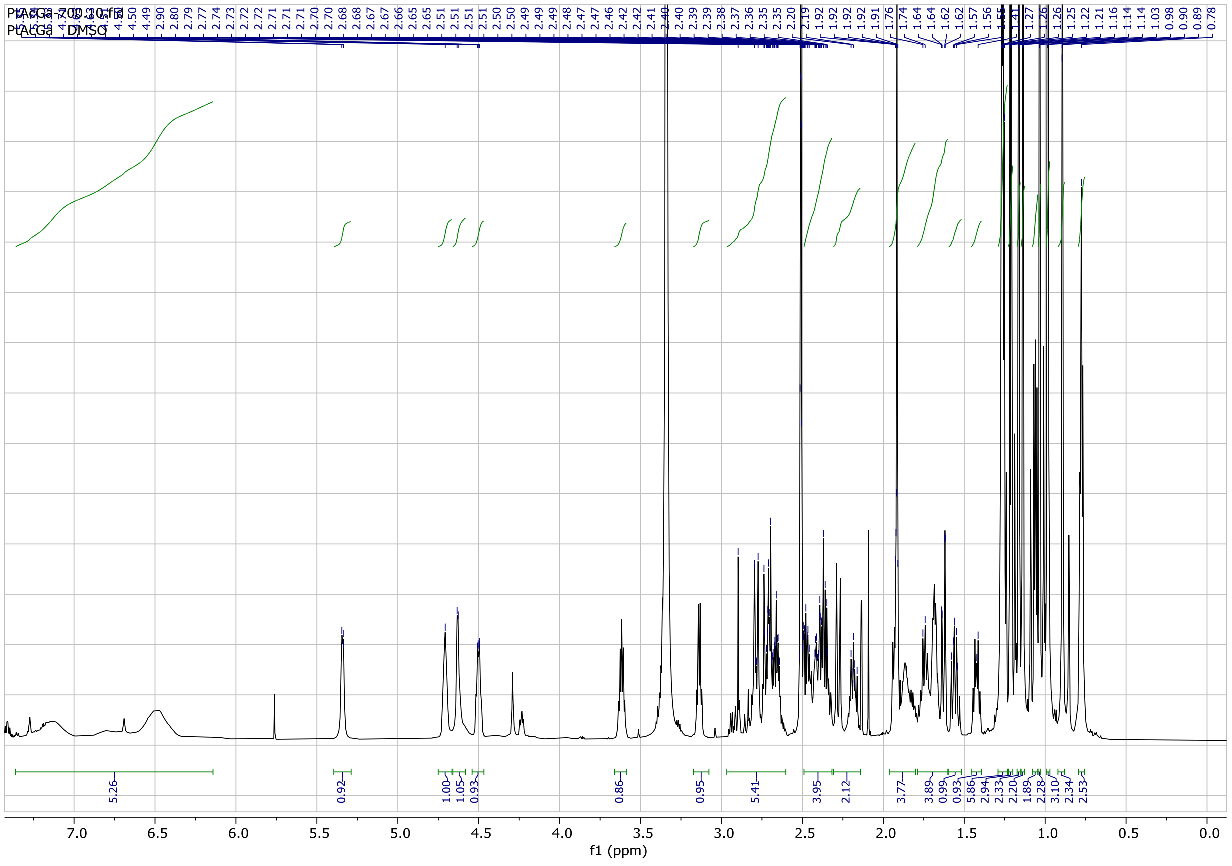

Figure S1-D. ^{1}H NMR spectra, enlargement of the 7-0 MHz range

© Gaiaschi et al.

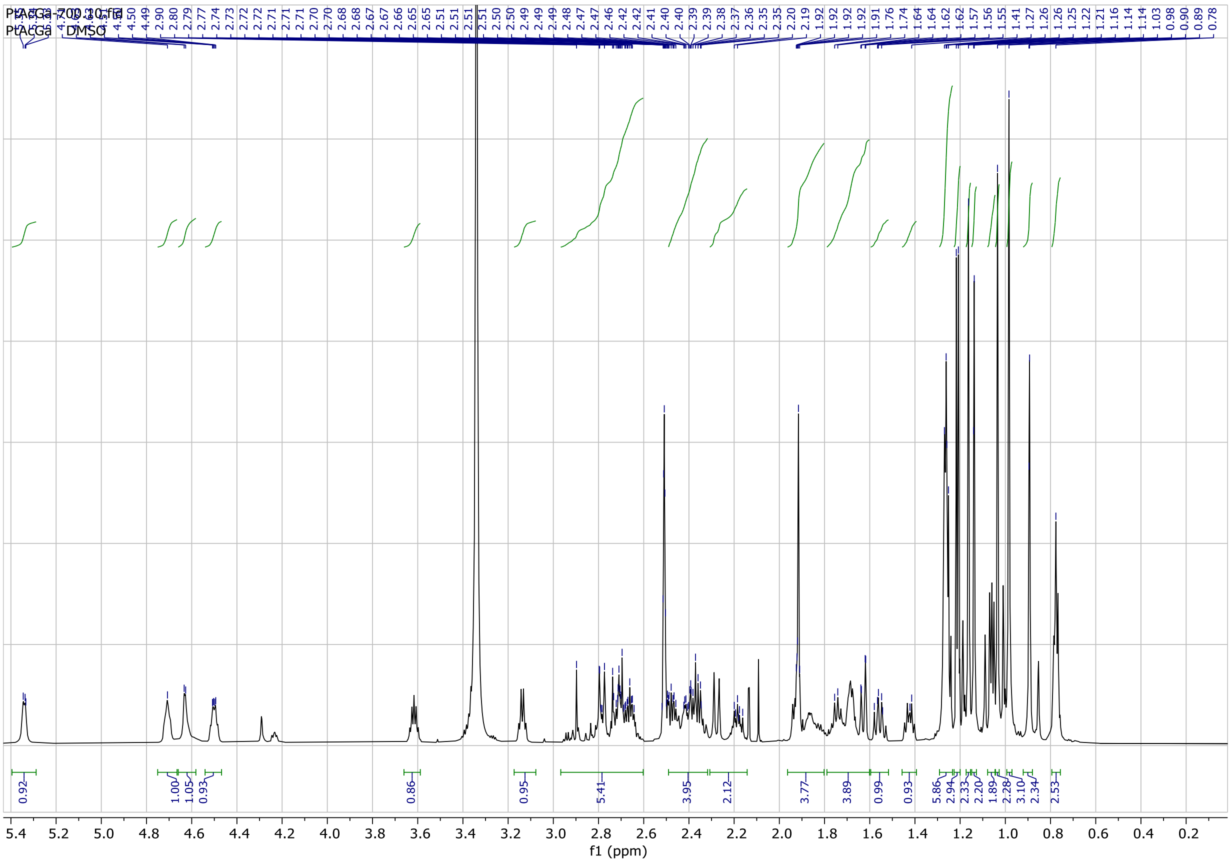

Figure S1-E. ^{1}H NMR spectra, enlargement of the 5.5-0.2 MHz range

ESI-MS: *m/z* calcd for $C_{32}H_{52}Cl_2N_2O_9Pt$ at $[M - H]^-$ 872.2625; found, 872.2619. Mass error: -1 ppm.

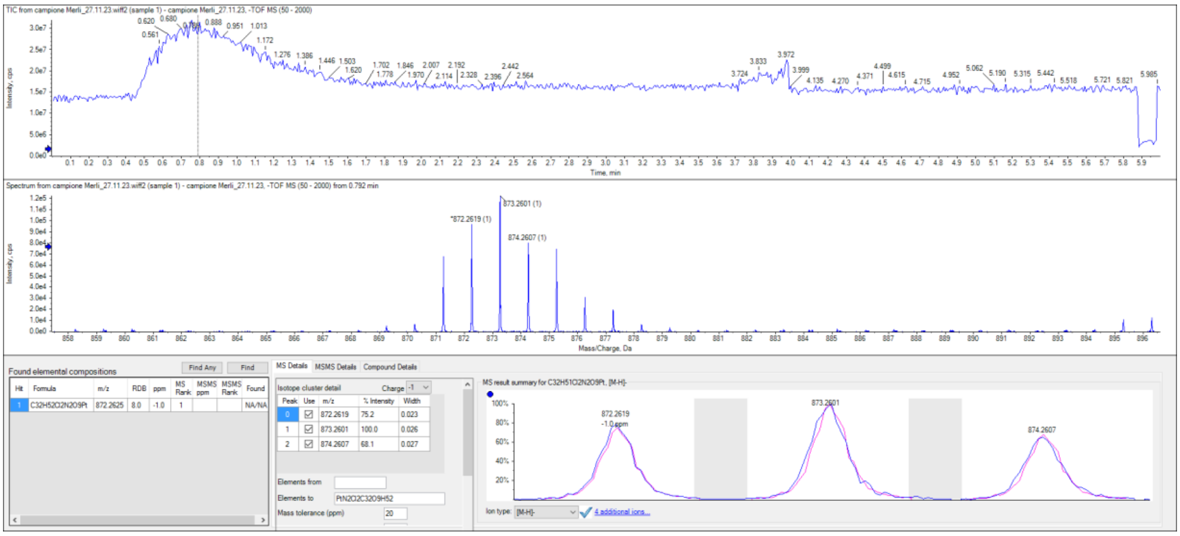

Figure S1-F. HRMS (ESI) spectra of Pt(IV)Ac-GA (predicted and experimental)

© Gaiaschi et al.
