# Supplementary material for: In Vitro Anti-Glioblastoma Activity of a Novel Pt(IV)-Ganoderic Acid A Conjugate
Source: Int J Mol Sci. 2026 Mar 18;27(6):2760. doi: 10.3390/ijms27062760 (PMC13026249; doi:10.3390/ijms27062760)
Supplement: Supplementary file 1 [file ijms-27-02760-s001.zip › Supplementary material S2.pdf]

Preliminary Exploration of Conformers and Spin States of Pt(IV)Ac-GA

All Gaussian 16 calculations reported here have been published in a dedicated repository [https://doi.org/10.19061/iochem-bd-6-557] on the *ioChem-BD* platform. Initial input structures were constructed using the Gaussview package (version 6.1.1).

While final calculations leading to logP prediction were carried out at the M11/MWB60/6-311+G(*d,p*) level of Density Functional Theory (see main text), all preliminary calculations reported here were carried out using the smaller 6-31+G(*d*) basis set for non-Pt atoms. This was to save computational time in these preliminary phases. The SMD solvation model was used throughout.

Spin States

Exploration of Platinum(IV) spin states was carried out by optimising model diacetate complex $[Pt(NH_3)_2(O_2CCH_3)_2Cl_2]$ in the singlet, triplet, and quintet states at the lower level of theory mentioned above, and performing frequency calculations to verify their nature as minima and estimate free energies. In this case, the chosen implicit solvent was dimethylsulfoxide (DMSO) as per our experimental work. The singlet state was found to be the most stable, with the triplet state having an estimated Gibbs free energy difference of + 39.1 kcal mol^{-1}, and the quintet state having an estimated Gibbs free energy difference of +85.1 kcal mol^{-1}.

Separate Conformational Exploration of the Organic Moiety and Platinum(IV) Complex

The only rotationally free part in the organic moiety is the 6C alkyl chain terminating with the carboxylate group: we directly assumed this to be in an extended *anti* conformation, and with the two methyl substituents *trans* to each other (see [https://doi.org/10.19061/iochem-bd-6-557] and **Figure S2-A**). No further conformational exploration was deemed necessary.

For the model octahedral platinum complex, after confirming the singlet state as the most energetically stable one, we continued to explore our model $[Pt(NH_3)_2(O_2CCH_3)_2Cl_2]$ complex through a series of optimisations to minima in implicit DMSO, and one relaxed potential energy surface scan in which one of the acetate ligands was rotated 360° about the Pt–O bond. Such calculations allowed us to establish that it is energetically more favourable to have: (1) both acetate C—C bonds perpendicular to the plane of $Cl_2Pt(NH_3)_2$; and (2) the unbound oxygen atom of each acetate ligand hovering halfway between the two nitrogen atoms (*i.e.*, with all C–O–Pt–N dihedrals ~±45°). For details, we again refer the reader to the calculations in [https://doi.org/10.19061/iochem-bd-6-557] and the conformation of the Pt complex in **Figure S2-A**.

Conformational Exploration and Preliminary Optimisation of full Pt(IV)Ac-GA

We then proceeded to explore the Pt(IV)Ac-GA in full, substituting one of the acetates in the $[Pt(NH_3)_2(O_2CCH_3)_2Cl_2]$ complex with a full ganoderic acid moiety featuring its terminal carboxylate in the same conformation. Continuing in implicit DMSO, but starting now also to repeat optimisations in implicit water and *n*-octanol, we further proved that it was more energetically convenient to bind the Pt complex to the carboxylate oxygen of ganoderic acid that was *trans* to the methyl substituent on the α carbon, rather than binding it to the *cis* carboxylate oxygen.

These final conformers of Pt(IV)Ac-GA optimised to minima at the M11/MWB60/6-31+G(*d*) level in implicit water and *n*-octanol served as the input for optimisation and frequency calculation at the (final) M11/MWB60/6-311+G(*d,p*) level. The resulting optimised conformer in water at this final level of theory is shown in **Figure S2-A** as an example.

© Gaiaschi et al.

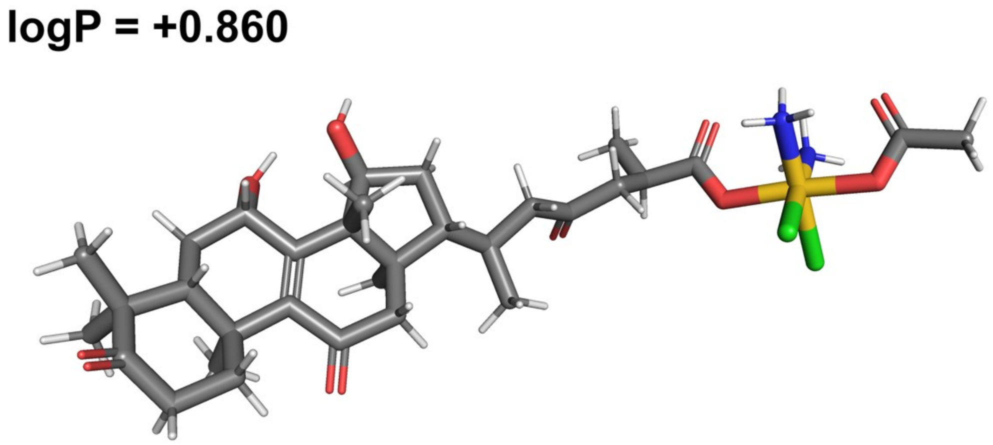

Figure S2-A. Low-energy conformer of the Pt(IV)Ac-GA complex optimised at the M11/MWB60/6-311+G(*d,p*) level of Density Functional Theory in implicit water, modelled *per* the SMD implicit solvation model. Optimisation of the same conformer in implicit *n*-octanol, resulted in very little structural differences (see [https://doi.org/10.19061/iochem-bd-6-557]). The predicted logP value was of +0.860. Key: grey: C; off-white: H; red: O; yellow: Pt; blue: N; green: Cl.

© Gaiaschi et al.
